# ‘Now that I am connected this isn't social isolation, this is engaging with people’: Staying connected during the COVID‐19 pandemic

**DOI:** 10.1111/bld.12478

**Published:** 2022-04-22

**Authors:** Natasha A. Spassiani, Mojca Becaj, Clare Miller, Andrew Hiddleston, Aaron Hume, Stephan Tait

**Affiliations:** ^1^ School of Health and Social Care Edinburgh Napier University Edinburgh UK

**Keywords:** COVID‐19, digital inclusion, disability, intellectual/developmental disability, technology

## Abstract

**Background:**

The COVID‐19 global pandemic has put adults with intellectual/developmental disabilities at greater risk of being socially excluded due to physical distancing. Technology has been looked at as a tool for adults with intellectual/developmental disabilities to stay connected, however, little is known about this topic. The purpose of this study was to explore how a grassroots disability organisation used technology to help adults with intellectual/developmental disabilities feel socially connected during the pandemic.

**Methods:**

Data were collected through questionnaires, attendance records, and field notes; and analysed through trend and thematic analysis.

**Findings:**

Four main themes emerged from the data: active leadership, mental wellbeing, technology/digital inclusion, and safety.

**Conclusion:**

These findings suggest that when participants overcome technological barriers they found it easy to socially connect online during lockdown.

## Accessible Summary

- This study helps us to understand how people with intellectual/developmental disabilities stayed connected with other people during the COVID‐19 pandemic.
- The study has been coauthored by people with intellectual/developmental disabilities to make sure their voices are included on this important topic about digital inclusion.
- The research project found that if given the opportunity people with intellectual/developmental disabilities can not only attend online events, but also organise and host their own online activities.
- The project also found that people with intellectual/developmental disabilities were able to build meaningful friendships online and feel empowered when leading their own online events.
- This study can help inform disability organisations on how technology can be used to support meaningful social inclusion for people with intellectual/developmental disabilities.

## 1 INTRODUCTION

The COVID‐19 pandemic has impacted people's lives in many ways. Society has had to learn how to stay connected while being apart. Social distancing has led to social isolation for many people. In the United Kingdom, people with disabilities were asked to self‐isolate due to their increased risk of getting the virus and dying from it (Turk et al., 2020). For this reason, many in‐person disability services were cancelled or put online. Providing online services has been a challenge because many people with disabilities, particularly people with intellectual/developmental disabilities do not have access to the internet or know how to use a computer (Caton & Melanie, 2016; Scholz et al., 2017; Shpigelman, 2021). This makes it very hard to access online services and support. Grassroots disability organisations work to include people with intellectual/developmental disabilities in society, but the pandemic has also made this hard. This study examines how a grassroots disability organisation has worked closely with people with intellectual/developmental disabilities to help them feel socially connected during the COVID‐19 pandemic through online activities and events.

### 1.1 Community participation and people with disabilities

Community living has led to a better quality of life for people with disabilities and has been related to improving physical, mental, and social health. Unfortunately, when compared to people without disabilities, people with disabilities are less likely to participate in the community, have meaningful relationships, and be socially included (Asselt‐Goverts et al., 2015; Simo˜es & Santos, 2016; Umb‐Carlsson & Sonnander, 2005; Umb‐Carlsson, 2008). Community participation for people with disabilities is difficult because of barriers, such as (1) accessibility, (2) lack of resources, (3) lack of social support, (4) the attitudes that people have about people with a disability being in the community and (5) policy/system barriers that do not support people with disabilities to be in the community (Hammel et al., 2008).

Being able to participate in the community is more than just being physically present. It needs the individual to actively engage with other people through social responsibilities and activities, like volunteering, working, or joining organisations/clubs (Cummins & Lau, 2003). Social inclusion has been defined by adults with disabilities as several different things:

- Being able to interact with community members who respond positively to them being in the community.
- Having access to community resources, such as facilities, venues and services (e.g., accessible, and affordable transportation).
- Having access to participate in opportunities of social inclusion (e.g., getting support from staff members to engage in community activities).
- Being accepted by community members and treated with respect (Abbott & McConkey, 2006).

Researchers have found that individuals with disabilities do not care about the number of community activities they participate in, for example, the number of times they go to the shopping centre. Rather, they are more interested in being part of community activities where they feel welcomed, respected by others, and able to engage with other people (Cummins & Lau, 2003). For this reason, it is important that research collects information from people with intellectual/developmental disabilities asking them about their experiences participating in the community, so that society knows how to better support people with disabilities living in the community.

Past research has found five key factors that affect how people with intellectual/developmental disabilities feel about how they belong to a community. These five factors are: (1) the person's self‐determination, (2) their social identity, (3) reciprocity (give and take), (4) valued contribution and expectations and (5) psychological safety (Milner & Kelly, 2009). Additionally, adults with intellectual/developmental disabilities reported feeling more comfortable to participate within the community when they are accompanied/surrounded by people they trust, such as their peers, support workers, and family members, they feel more comfortable engaging in social settings with fewer people and express a need for mental/emotional safety when engaging in community activities. People with intellectual/developmental disabilities tend to want to look for opportunities for community participation as a means to ‘prove' to the public that they are capable, valuable and contributing members of the community (Milner & Kelly, 2009). Grassroots disability organisations have played a very important role in making sure that people with disabilities are included in the community.

### 1.2 Grassroots disability organisations

Grassroots disability organisations, which are organisations that involve people with disabilities coming together to advocate for the human rights of people with disabilities, have played a very important role in ensuring people with disabilities are fully included in all parts of community living. Grassroots disability organisations have helped to break down false views about people with disabilities. These organisations have helped show society that people with disability can achieve the same things as nondisabled people when they are given equal and fair opportunities (Dowse, 2001). Grassroots disability organisations have had a very important role in helping society understand that people with disabilities are not to be treated as charity cases, but are humans that deserve the same opportunities and human rights as nondisabled people (Kemple et al., 2011). These organisations challenge the stigma and stereotypes about disability and help to show society that people with disabilities are self‐determined individuals, regardless of the constant discrimination and oppression they experience from society (Chappell et al., 2001). These organisations help to provide people with disabilities opportunities for inclusion, employment, independent living, education, and access to resources, such as technology.

### 1.3 Technology and people with Intellectual/Developmental disabilities

Technology has provided new ways to participate in daily life and build social relationships (Ramsten et al., 2020). The majority of research about technology involving adults with intellectual/developmental disabilities has examined:

- Access to the internet,
- Common barriers and opportunities they may face when using the internet (Caton & Chapman, 2016; Chadwick et al., 2013),
- Risks of being bullied or taken advantage of online (Normand & St‐Louis, 2016),
- How to support people with intellectual/developmental disabilities to use the internet safely (Seale, 2014).

People with intellectual/developmental disabilities are less likely to have access to the internet. For example, people with intellectual/developmental disabilities living in Europe are 62% less likely to have access to the internet (Scholz et al., 2017). There are a number of reasons that affect why people with intellectual/developmental disabilities have limited access to the internet. Some of these reasons are; (1) the individual's cognitive impairment, (2) education, (3) politics, (4) economics, (5) education, (6) training and support to learn how to use the Internet and (7) nondisabled people's attitudes about people with intellectual/developmental disabilities using the internet (Chadwick et al., 2013).

Researchers (Caton & Chapman, 2016) found common barriers to accessing the internet for people with intellectual/developmental disabilities. Some of the barriers they found are (1) safety, (2) cyber language/etiquette, (3) having access to internet connection, (4) having access to proper equipment like a computer or internet modem and (5) the internet itself not being accessible for people with intellectual/developmental disabilities to use. Other research has found that people with intellectual/developmental disabilities commonly have issues with how to work technology, reading and abstract thinking (Lussier‐Desrochers et al., 2017).

Research has shown there are also positive aspects of people with intellectual/developmental disabilities having internet access, such as (1) making friendships, (2) enjoyment and (3) developing self‐esteem and self‐identity (Chadwick et al., 2013). Research has also found technology can help young adults with intellectual/developmental disabilities participate in activities in their homes and in their communities, which has been shown to help people with intellectual/developmental disabilities become more independent in their daily life (Ramsten et al., 2020).

### 1.4 Technology, COVID‐19, and people with Intellectual/Developmental disabilities

The COVID‐19 pandemic has forced people with intellectual/developmental disabilities to use technology to stay connected. However, research has shown the pandemic has highlighted that people with intellectual/developmental disabilities who do not have access to technology and digital services (e.g., the internet) are being left behind during this crucial time (Chadwick et al., 2022). Research has found during the pandemic people with intellectual/developmental disabilities were not able to use technology to stay socially connected due to lack of digital literacy skills (knowing how to use technology) and confidence in using technology to participate in online social activities (Lake et al., 2021; McCausland et al., 2021), needing support of someone to help use technology which was not always available (Datlen et al., 2020; Lake et al., 2021), protection and security concerns about the internet being a safe place (Power et al., 2021; Rawlings et al., 2021), the cost of digital devices (Lake et al., 2021), having no internet connection (Power et al., 2021) and digital communication not being accessible for individuals with sensory needs (Rothman, 2021).

Research shows during the COVID‐19 pandemic people with intellectual/developmental disabilities were (are?) more likely to be able to use technology if they had prior experience with using technology (Amor et al., 2021; Rawlings et al., 2021) and had access to technical support, usually from caregivers or the individual's personal support network (Amor et al., 2021; Power et al., 2021; Rawlings et al., 2021). A UK study that took place during the COVID‐19 pandemic found that 92% of participants with intellectual/developmental disabilities use the internet at home. This study found people with intellectual/developmental disabilities most commonly used the internet during the pandemic to video call their family and friends, go on social media, and watch television shows and films (Flynn et al., 2021).

### 1.5 How COVID‐19 has impacted people with Intellectual/Developmental disabilities

The global pandemic of COVID‐19 has impacted the lives of everyone. This is particularly true for people with disabilities as they are at great risk of not only contracting COVID‐19, but also of getting very sick from it and requiring hospital treatment (Landes et al., 2020; Turk et al., 2020). For example, people with Down's syndrome may be more vulnerable to suffering from serious COVID‐19 complications (De Cauwer & Spaepen, 2021). People with intellectual/developmental disabilities ages 0–17 years are 20 times more likely to get COVID‐19 (Turk et al., 2020). People with intellectual/developmental disabilities are 2.5 times more likely to die of COVID‐19 (Turk et al., 2020). For these reasons, physical isolation has been very important for people with disabilities, however, this may have serious effects on their mental health (Courtenay & Perera, 2020; Javed et al., 2020). Services that are typically accessed by people with disabilities such as day services and day activities had to stop, leaving many people with intellectual/developmental disabilities unable to access their social networks. For this reason, technology has been explored as a tool for participating in daily activities such as work meetings and social gatherings which can be all conducted online (Caton & Chapman, 2016; Shpigelman, 2018, 2021).

Disability organisations and services have had to act quickly to provide online services and support for people with intellectual/developmental disabilities to ensure they are able to access care and social connections. There is a need for technology training and support for service providers to ensure people with intellectual/developmental disabilities can be digitally included (Chadwick et al., 2022). However, there is limited research done in this area on disability organisations and service providers supporting people with intellectual and developmental disabilities learning to use technology to participate in digital platforms.

### 1.6 The purpose of this research study

The purpose of this study is to explore how a grassroots disability organisation used technology to help adults with disabilities feel socially connected during the COVID‐19 pandemic.

### 1.7 Social justice and inclusive research

This study study has looked to social justice theory to understand how people with intellectual/developmental disabilities can be socially included during the COVID‐19 pandemic. When we think of including people with intellectual/developmental disabilities in society we typically think of them being physically included with a passive role in society (e.g., sitting around the table but unable to contribute). The social justice theory tells us that for people to be included in society they need to be meaningfully participating in society and most importantly, feel empowered (Gidley et al., 2010). For example, people with intellectual/developmental disabilities having leadership roles, being employed and developing meaningful relationships in the community.

Research about people with intellectual/developmental disabilities should actively involve people with intellectual/developmental disabilities to ensure research findings are understood and presented to the wider community (Walmsley, 2001, 2004). We wanted to highlight the importance of having the voice of people with intellectual/developmental disabilities present in this research article. For this reason, self‐advocates with intellectual/developmental disabilities were key members of the research team. More specifically for this research project, self‐advocates contributed to analysing the data helped bring research findings to light and participated in writing the introduction, results and discussion section of this paper. Members of the research team who did not have intellectual/developmental disabilities helped to facilitate the research process in an accessible manner. We wrote this study in accessible language because we wanted our research to be understood by a wider audience, such as other people with disabilities and disability organisations.

## 2 METHODS

### 2.1 Sample and characteristic of the grassroots disability organisation

This study looked at a small UK based grassroot disability organisation that creates social opportunities for adults with intellectual and developmental disabilities in and around a large city in Scotland. The organisation has a membership of over 900 adults with disabilities. Approximately 80% of members have an intellectual/developmental disability. For this study, 30 members with intellectual and developmental disabilities participated and were not hand selected. Participants' ages ranged from 18 to 56 years and there was an equal number of men and women who took part in the study. Members with more complex needs were not represented in this study.

Before the COVID‐19 pandemic, the organisation hosted all of its events in person in the community, utilising activities and places that were on offer to the whole population. It hosted on average 15 events per month doing things such as cocktail making, life drawing, and clubbing in a very popular local nightclub. Since the beginning of the COVID‐19 lockdown, this organisation had to move all their operations online. They now use technology to offer the same sense of community that their members enjoyed before the pandemic.

To deliver an engaging, fun and accessible online events programme, the staff team worked closely with the membership and was actively seeking feedback from people with disabilities attending events. The inclusive approach ensured that members were able to suggest and lead on activities, boost their confidence and make a difference in the lives of those who were able to take part. The organisation had to work hard to actively help members gain access to the online opportunities that had been created. Staff have coached members step‐by‐step either through email, Facebook messenger or phone. Step‐by‐step guides have also been featured in the newsletter and over the pandemic the organisation has created and built up a video tutorial library which features adults with disabilities using the various online platforms. Over the pandemic the Ambassadors and sessional workers have gained digital skills and the confidence to support other members with technical issues.

Since April 2020, the organisation set up a closed Facebook group for its members, allowing a safe space for adults with disabilities to chat and keep connected during the pandemic. This closed community group is open to all members and moderated by the organisation's staff. The main moderating job is cross checking with the membership database, making sure that people requesting to join the group are members of the organisation. Employed staff with disabilities have worked with a Self‐selected group of around 30 members who actively develop and produce the content for the page. They meet weekly to discuss what activities members would like to see and plan for the week ahead. In May 2020, the organisation started trialling Zoom events, as they were aware not all of their membership was on Facebook. Through Zoom, the organisation hosted 15–18 events for its members each month in addition to 20–35 Facebook activities which were created by online community contributors. The organisation continues to be guided by the members in choosing what events to host and they regularly bring in external experts to keep broadening experiences. The online content continues to grow in diversity and depth as members grow in technical confidence.

Online events have included comedy nights, quiz nights, seated exercises, sing‐alongs, dance classes, puzzles, horoscope readings, and workshops. The organisation also transferred its club nights onto Zoom. This is now a monthly event that uses Zoom to have different breakout rooms so that there is a ‘bar area' where members talk, and a ‘dancefloor' where the DJs, all of whom are adults with disabilities, do their sets and members can listen to the music and dance.

### 2.2 Data collection

Ethics approval was granted by the community organisations. Members gave written consent for data to be collected by the grassroots organisation. Data for this study were collected through attendance records, questionnaires, and field notes. The organisation had designed and collected the data for this study and then asked researchers to join the team to help them analyse the data collected.

### 2.3 Questionnaire

The questionnaire was developed by staff and questions focused on member with intellectual/developmental disabilities' satisfaction with online activities, and general feedback about online engagement. The purpose of the questionnaire was to gain a better understanding of the experiences of people with intellectual/developmental disabilities who were engaging with the online activities so the organisation would be able to better support its members. The questionnaire included multiple choices, short answers and yes and no questions. An example of questions asked where: *Did you feel encouraged to contribute to the Facebook group? What did you think would make the Facebook group better? Did you learn new digital skills?*

The questionnaire was distributed via email, Facebook and Zoom group chat to all 30 members of the planning group (all of which have intellectual/developmental disabilities) and filled out by those who chose to do so. The data were collected at 3 months post moving online (June 2020), at 6 months (September 2020), 9 months (December 2020), and at 12 months (March 2021).

### 2.4 Attendance records

Number of events each month and member participation at all events was collected using Zoom and Facebook data.

- For in‐person events pre‐Covid, the number of attendees was counted by who turned up at the time and place of the event.
- For Zoom events, the number of attendees was counted from who joined the event on Zoom.
- For Facebook events, the data came from the number of people who watched a Facebook live, or the number of people who participated in an activity such as a quiz or a photo challenge.
- The number of members who hosted events was also counted each month, as well as the number of members who created other forms of content for the Facebook page.

### 2.5 Field notes

Field notes were gathered from March 2020 to March 2021 during online events. Staff took notes from either typed conversations taking place in the chatbox or took notes of verbal conversations had during the events. Field notes were collected this way as it was a more natural and would therefore lead to more meaningful responses. Members with intellectual/developmental disabilities were aware that field notes were being taken. Field notes were taken for the purpose of assessing connection and empowerment through online platforms as well as to assess and increase accessibility of online events. All data collected were anonymized for confidentiality.

### 2.6 Analyses

Attendance records were analysed through descriptive statistics. Field notes and questionnaire data were analysed using thematic analysis (Corbin & Strauss, 1990). The data was originally analysed by two staff members and organised into manageable parts. Once this had been completed, the research team (which included two staff members, three individuals with intellectual/developmental disabilities, and a university researcher) coded for main themes as a group. The data were checked for consistency and any differences in coding were discussed. The research team worked together to go over the themes to make sure everyone was in agreement with how findings were interpreted (Patton, 2002).

## 3 RESULTS

### 3.1 Trends in attendance and opportunities

From the data collected for overall online event attendance over the first 12 months (March 2020 to March 2021) of the COVID‐19 pandemic, attendance at all events increased by 69.8%, compared to the in‐person events in the community the year before.

### 3.2 Attendance of Zoom events

Over a period of 12 months, from March 2020 to March 2021 the average Zoom event attendance was 19 people. This number is greater than in the previous year when the organisation was hosting in‐person events, where the average attendance at each event was 10 people. In February 2021, the average number of people attending Zoom events had increased from 15 (January 2020) to 21 people. In total between March 2020 and March 2021, 3334 people attended Zoom events, however, it is important to note that some of these individuals may have attended multiple events.

### 3.3 Attendance of Facebook events

Over a period of 12 months, from March 2020 to March 2021 the average event attendance during Facebook events was 10 people. Facebook events showed an impressive engagement of 3941 attendances over the course of 12 months. Additionally, since the organisation created the Facebook group, 30 members with disabilities created and led 415 Facebook events.

### 3.4 Geographic area

Before the move to an online platform due to the COVID‐19 pandemic, membership data showed that all members lived in a localised geographic area in Scotland. During the pandemic people joined from further away, not just from other areas of Scotland, but also from other areas of the UK such as Wales.

### 3.5 Themes from analysis of questionnaire & field notes

From analysis of the questionnaire and field note responses, four themes appeared. These themes are:

- Active leadership
- Mental wellbeing
- Safety
- Technology and digital inclusion

### 3.6 Active leadership

The indicators for active leadership were the number of adults with disabilities leading online member‐led initiatives. This increased by 56%, from 30 people (March 2020) to 54 people (March 2021). Members stated that taking on leadership roles helped them feel more confident and improved their communication skills. One member stated, ‘I come along to the weekly planning meeting to get ideas and I work on a photo challenge every 2 weeks for the Facebook group. I really enjoy it; I am getting to know people through this and I am becoming more confident and able to communicate better.’

Active leadership was noted in the short answer replies of the questionnaires. When asked what would make the organisation work better online members at both timepoints spoke about wanting more people to join, but at 6 months people spoke in more depth about how that could happen—‘More events on Zoom instead of Facebook since not everyone is on Facebook and you only need an email account for Zoom‘.

At 6 months adults with disabilities also spoke about how they could help personally. For example, one person stated, ‘I could work alongside members and help them to learn to do events especially if they don't feel comfortable doing it themselves’. Field notes further added to this idea of active leadership with members speaking about how important it was for them to help others—‘events have made me have a purpose, pass the time by making differences to others’. Members also spoke about feeling that they were learning from each other—‘It has been great—we have new fresh ideas now… the more we work together, the more we learn.’

At 9 months, members started passing on projects they started so others could also step up and try leading initiatives. One person said ‘I would like to work with other people in the future and welcome other people on board. I see it as an evolving project, perhaps passing things over when someone suitable comes along’. Ambassadors started chairing the meeting and offering weekly opportunities within the online community. An Ambassador reflected ‘Through my work as an Ambassador, I want to inspire other people with learning disabilities to believe that a paid job is a possibility and can be a reality for them too.’

In March 2021, the community celebrated 365 days online with an online festival and some member‐led initiatives which opened new opportunities for members to be involved in more than just one way. A member who was doing quizzes every other week during the pandemic reflected ‘That whole journey has been incredible—we felt a lot of support from members, and the members wanted to get involved with the quizzes, so people started working with us to bring forward their ideas or certain topics—such as music quiz, or horrible histories quiz. I have to say that people are learning on quizzes as well, some of them would not even think of the facts before. It has been a big learning curve for me and the members – it could take about a week for me to make a quiz, with different facts, questions, answers, research and members' input. It was amazing, those quizzes are a total buzz! What is good about those quizzes is that now there are opportunities for other members to host the quiz and who knows, it could even become their new thing!’

### 3.7 Mental wellbeing

Feeling connected to others and the impact on mental wellbeing is another theme found in this study. Members highlighted how online events helped them with their mental wellbeing. One person stated ‘If I didn't have these I feel my mental health would have been badly affected. I have events to look forward to instead of nothing.’ Members also highlighted how staying connected with other people through the organisation and the online events was a key support for them during the pandemic. One person said ‘seeing people's faces and connecting with people has been really important.’

Members spoke about how being an adult with a disability and having pre‐existing health conditions living through the pandemic had a particular impact on mental wellbeing. One person said, ‘Especially during the lockdown, feelings are quite high, people are feeling under pressure, especially all of us who are shielding at the moment’ Members spoke about how important the online events and opportunities were for managing their mental wellbeing during the pandemic. For example, one individual said ‘If I didn't have {the Facebook page} I'd be lost, no quizzes or online events either. It's kept me occupied and not worried about the current situation in the world.’

Members also spoke about how being open and connecting to others helped them become more emotionally resilient. One person said ‘We find inner strengths with these groups’. The questionnaire data showed that at 3 months into lockdown 47% of members stated that they had made new friends, this rose to 70% at 6 months, 87% at 9 months and 76% at 12 months Feeling connected and feeling part of a group with shared experiences was important for personal growth—people spoke on behalf of each other ‘We find inner strength with these groups’, showing that there was a sense of growing together and supporting each other through it. The increased sense of online connection was seen as something positive to have come out of the COVID‐19 situation. As one person said ‘COVID isn't great, but it has brought us together! (…) For us, lockdown has been pretty awesome!’

### 3.8 Safety

Since starting events online, there has been a high uptake for events that encourage deeper conversation such as the regular *Let's Talk About Sex* events which had on average 20 attendees at each session. During these sessions members opened up about things on a much deeper level than was happening\during in person sessions before the pandemic. Members spoke regularly about how ‘having a safe space’ to talk is really important. Members also spoke about feeling that they could try out online events such as the club night because it was safer for them than going to a real club. For example, one person said ‘My autism is not obvious. Online there are safe spaces, at {the club} there is no safe space to go. Here at home, you can take time out and you can click on the call again.’

### 3.9 Technology and digital inclusion

Being digitally connected is another theme found in the current study. Members spoke about how being digitally connected during lockdown helped them to stay connected with others. For example, one member said—‘If I wasn't digitally connected… at home I would just be fiddling my fingers.’ Another member stated—‘The Facebook events and Zoom events have been essential for me to feel connected to others’.

Responses to the questionnaire suggest that members learned about technology through being part of the organisation. At 3 months 100% agreed or were neutral on the statement ‘I learned new things about technology’, and at 6 months 90% still responded this way, 87% at 9 months 76% at 12 months. These data are backed up by field notes—‘I've learned so much helpful information about technology and computer apps.

One individual spoke about how the organisation helped them overcome social isolation during the pandemic: ‘I was staying with my parents and they couldn't help me to get set up with zoom. They call it social isolation ‐ but now that I am connected this isn't social isolation, this is engaging with people.’ In the first 6 months of the pandemic, the organisation managed to digitally connect 26% of its membership through their community Facebook group. The remaining 74% of this population is still not connected. Field notes highlight how access to technology has given people opportunities for inclusion that were not there before. One individual stated, ‘being digitally connected has helped me make new friends.’

## 4 DISCUSSION

The purpose of this study is to explore how a grassroots disability organisation used technology to help adults with disabilities feel socially connected during the COVID‐19 pandemic. In the past, people with intellectual/developmental disabilities have been largely excluded from being connected online. Disability services and organisations mostly meet in‐person, and this was one of the main ways people with intellectual/developmental disabilities were able to socialise and connect with society. Since the pandemic started, people with intellectual/developmental disabilities have had to self‐isolate to protect themselves from getting sick. This meant not going out into the community, not being able to hang out with family and friends, quitting their jobs and volunteer work, or going to school to keep themselves safe. Some organisations and services have switched to being online to keep people with intellectual/developmental disabilities socially connected.

Our study found that adults with intellectual/developmental disabilities were able to meaningfully participate online and felt socially connected through their computer screen. The grassroots disability organisation in our study was able to create a digital community for its members with intellectual/developmental disabilities, offering events and activities that members would typically participate in before the pandemic. For example, disco nights, comedy nights, quiz nights, seated exercises, sing‐alongs, dance classes, puzzles, horoscope readings, and workshops. From our study we found a few key points:

1. Since moving online, the organisation saw a large increase in active participation with more adults with intellectual/developmental disabilities attending events.
2. Since moving online, adult with intellectual/developmental disabilities reported increased self‐determination and personal growth.
3. Since moving online more adults with intellectual/developmental disabilities took on active leadership roles and felt that the organisation became a community.

Below we talk about each of these points in more detail.

*1. Since moving online the organisation saw a large increase in active participation with more*adults with intellectual/developmental disabilities attending events.

Some reasons are follows.

*(1a) Easier access to online events*

Adults with intellectual/developmental disabilities were able to be included because being online allowed for easier access to events and activities especially during lockdown. Members with intellectual/developmental disabilities who might usually be unable to attend events in the community were able to come to events online. The organisation was able to offer opportunities to a wider variety of members—members who were previously unreachable. Even those who are normally able to access the community are often restricted by other barriers such as money, support needs, and transportation (Hammel et al., 2008; Marks & Heller, 2003; Spassiani et al., 2019). Before the COVID‐19 pandemic forced the organisation to go fully online, many members would choose only one or two events to attend each month. The move online has meant that members with intellectual/developmental disability can now choose to come to multiple events each week.

*(1b) Digital connection and activity were important to mental wellbeing*

The pandemic left people without their usual day centres and activities. In many cases, it left people with intellectual/developmental disabilities without services that supported their mental wellbeing. Members were able to use online events to fill up their time and feel connected. Past research says that people with intellectual/developmental disabilities are looking for community activities where they feel welcomed, respected, and can engage with others (Cummins & Lau, 2003). Our study found that by creating a digital community, adults with intellectual/developmental disabilities may have felt connected and supported. Adults with intellectual/developmental disabilities found that being involved with the events and connecting with others helped them through the difficulties of the pandemic. As a result, they continued to do more and get more involved.

*2*. Since moving online, adults with intellectual/developmental disabilities reported increased self‐determination and personal growth.

Some reasons for this may be due to:

*(2a) Increased opportunity to access events*

By offering more online events, there may be less pressure for individuals to participate during events. With more events being offered online, members with intellectual/developmental disabilities had the time to take smaller steps, with more opportunities to try, fail and try again, therefore building up skills and confidence more naturally. They are able to spend more time with a larger number of people so that they are able to find out which other people have similar interests and build true and meaningful relationships. Other research has found that adults with intellectual/developmental disabilities are able to build positive relationships online (Holmes & O'Loughlin, 2014). For example, people with intellectual/developmental disabilities can build online relationships that can have the same benefits as seeing people in person (Holmes & O'Loughlin, 2014) and help them feel less lonely (Kydland et al., 2012). Past research has found that it does not matter how many community events an individual with an intellectual/developmental disability attend, but rather it is more important the individual feels included (Cummins & Lau, 2003). Our study found that having many online events available allowed individuals with intellectual/developmental disabilities the opportunity to attend many events and familiarise themselves with how to socially engage in the digital world, which was seen as a positive.

*(2b) Increased sense of safety meant people felt more able to take chances/risks*

Past research has talked about how the internet may not be a safe place for people with intellectual/developmental disabilities because of worries about privacy, bullying, and how someone should behave when talking to people online. For example, considering what pictures or videos are safe to share with someone you meet online (Holmes & O'Loughlin, 2014; Löfgren‐Mårtenson, 2008). When talking about online safety it is important to also talk about how online events can make people feel emotionally and physically safe. Being able to stay at home for events takes away many unknown factors. For example, people know their environment, people do not have to worry about transportation, and people know they can easily get out of a situation if it starts to feel uncomfortable. This allows members to put themselves out of their comfort zone slightly because they are in a safe environment (i.e., their home). This might be particularly true for members with sensory processing issues, specific health issues or anxiety stemming from past trauma. Additionally, since membership to the organisation is open to any adult with a disability, people can interact with others of varying levels of life experience and disability while being in a safe environment. This means that people can see what others are doing or have done in their lives and can encourage people to try different things. The online safe space that the disability organisation created allowed individuals with intellectual/developmental disabilities the opportunity to have meaningful conversations with their peers that did not include their carers or parents. This is something that members with intellectual/developmental disabilities found particularly valuable.

*3. Since moving online more adults with intellectual/developmental disabilities took on active leadership roles and felt that the organisation became a community*.

Some reasons for this are:

*(3a) Working together to strengthen the sense of community within the organisation*

Members valued the organisation and had a sense of purpose to help the organisation adapt and succeed through the pandemic. Being thrown into a difficult situation gave everyone a sense of togetherness. Everyone, including the staff, were at a disadvantage and felt driven to help the organisation to ensure adults with intellectual/developmental disabilities could access their services during this difficult time. The organisation was forced to adapt the way they provided services to its members. In adapting their services, staff, and members worked closely together to provide an accessible online community for adults with intellectual/developmental disabilities. Seeing how well the organisation adapted and was able to keep providing services gave members a sense of shared pride because they had an active role in the organisation's success. This sense of pride may have helped strengthen the community as adults with intellectual/developmental disabilities felt a sense of ownership towards the community organisation. Communities that are connected will help strengthen how marginalised groups (like adults with intellectual/developmental disabilities) feel about making their own choices and being independent. The COVID‐19 pandemic, although negative in many ways, has also provided communities (like disability organisations) the opportunity to increase their strength, connectivity, and resilience (Russell, 2021). This study is an example of how a community organisation took something negative and used it to strengthen the sense of community within the organisation.

*(3b) Active role modelling may lead to more people feeling confident to take leadership opportunities*

Within our study, 30 members with intellectual/developmental disabilities created and led all the online Facebook events. Depending on individual preference, members worked independently or in groups to develop online content. The organisation encouraged and supported individuals to take on leadership roles through working with the individuals' own needs. The organisation fostered opportunities and created a platform that allowed for ideas to be brainstormed and developed as a group, where members felt inspired to contribute to content development. Results showed that adults with intellectual/developmental disabilities attended multiple events throughout the year. Repeat attendance would suggest that people enjoyed the events created and led by adults with intellectual/developmental disabilities and wanted to come back again. This finding builds on past research that has typically looked at supports and barriers of people with intellectual/developmental disabilities being digitally included (Caton & Chapman, 2016; Lussier‐Desrochers et al., 2017). However, our study has found that when people with intellectual/developmental disabilities are supported by a disability organisation they are able to feel empowered to not only participate in social events online, but to take on active leadership roles on digital platforms.

The organisation provided its members with opportunities to take on active leadership roles. This provided adults with intellectual/developmental disabilities more opportunities to see people they can relate to being leaders. Adults with intellectual/developmental disabilities were able to see that leadership roles can be possible for themselves as well. The more an individual sees this, the more likely they are to feel capable themselves. The more leadership roles an individual takes on, the more they feel capable of trying something else. Those in leadership roles wanted these opportunities to be available to others to help them succeed. This is similar to past research finding people with intellectual/developmental disabilities are interested in leadership opportunities to demonstrate to the community that they are independent and valuable members of society (Milner & Kelly, 2009). It is very important that leadership opportunities within an organisation are provided to help facilitate change in the community (Russell, 2021). For example, by leadership roles being given to adults with intellectual/developmental disabilities within the organisation, this can lead to adults with intellectual/developmental disabilities feeling empowered to take on other leadership roles in society.

Non‐profit organisations are good at being creative to solve problems, for this reason, service providers and governments should look to non‐profit organisations to learn how they cope with crises and move forward. Community participation is key to designing and delivering new services. Community leadership helps to bring creative and new ways to look at problems in society (Russell, 2021), which has been particularly true during the time of the COVID‐19 pandemic, where community organisations have worked closely with their members to identify solutions to support them through this difficult time (Marston & Miles, 2020). Past research has stated that the COVID‐19 pandemic has highlighted the importance of disability service providers to be educated about digital inclusion and to educate and support people with intellectual/developmental disabilities in being able to use technology (Power et al., 2021; Scheffer et al., 2021). Past research has also stated with digital inclusion we need to be very cautious that we are not excluding people with intellectual/developmental disabilities who may not know how to use technology (Setchell & Torres, 2021). Our study findings support past research and demonstrates how effective disability organisations can be in educating and supporting people with intellectual/developmental disabilities in staying digitally included.

### 4.1 How might this study be useful post‐pandemic?

The findings of the study may be useful even after the pandemic is over because it shows how important digital inclusion is for people with intellectual/developmental disabilities. Organisations should work towards offering online opportunities for people with intellectual/developmental disabilities to connect with other adults who may not be able to physically access the community. Online opportunities act as stepping stones into the community. In other words, online interactions can allow people with intellectual/developmental disabilities to build friendships more gradually so that when they do have their time in the community, they may be more aware of who they want to spend time with. This makes the time they can spend with others in a physical setting more likely to be meaningful. Additionally, disability organisations should consider allowing its members with intellectual/developmental disabilities to have active leadership roles hosting online events to help develop confidence, independence, and overall mental wellbeing.

In our study, the organisation provided opportunities for members to develop and host online events. This led to adults with intellectual/developmental disabilities feeling empowered to be given such an important job. It also helped other members see what their peers are capable of and encouraged others to take on leadership roles, like for example, hosting their own online event for the organisation. This is an important finding that disability organisations and services should think about to help support the mental wellbeing and personal growth of adults with intellectual/developmental disabilities.

Every research study has limitations. This study is limited by the small number of people who completed the questionnaire (20 adults with intellectual/developmental disabilities). This means that our findings are limited to this small group. Future research could look at having a larger group of people with intellectual/developmental disabilities from different grassroots organisations complete the questionnaire so we can get to know the experiences of more people. Additionally, the questionnaire that was used in this study was developed by the organisation and were specific to the organisation's interest. Therefore, this questionnaire may not be relevant to use by other organisations. Our study does not address the number of adults with intellectual/developmental disabilities who do not have access to technology and who are being digitally excluded because of this. Further research should look at how we can make the digital world more inclusive for all people with disabilities.

Self‐advocates with intellectual/developmental disabilities played a key role in the data analysis and writing of this paper. Some of the self‐advocates did not have prior experience with conducting research so it was very important that other members of the research team worked closely together to ensure everyone understood the process. We worked together as a team and had much discussions around the results and what they mean for the intellectual/developmental disability community. Writing this paper as a team took us almost a year to complete as a group because we wanted to make sure it was an inclusive and meaningful process for everyone involved. We do not think this is a limitation, but rather a reasonable accommodation to ensure people with intellectual/developmental disabilities have an active voice in research that is about them (Walmsley, 2001, 2004),

## 5 CONCLUSION

The purpose of this study is to explore how a grassroots disability organisation used technology to help adults with disabilities feel socially connected during the COVID‐19 pandemic. From our research project, we have shown how a small disability grassroots organisation was able to thrive during the pandemic by primarily adapting and actively involving members with intellectual/developmental disabilities to design and deliver services. The study demonstrated that people with intellectual/developmental disabilities can learn how to use technology and digital platforms (such as Facebook and Zoom) during a global pandemic provided they receive the proper support. This highlights the important role the grassroots disability organisation plays in making sure they can provide digital education to its members.

Our study also shows that through the support of the grassroots organisation, individuals with intellectual/developmental disabilities can be empowered to take on leadership roles and independently organise and lead online events. These important findings can be used to help policy makers and funding agencies understand the important role that disability grassroots organisations play in helping to empower people with intellectual/developmental disabilities. Future research should examine how people with intellectual/developmental disabilities can be supported and empowered to take on leadership roles in the digital world.

*From our study we have come up with 6 take away points for service providers who supporting adults with intellectual/developmental disabilities*:

1. Adults with intellectual/developmental disabilities are capable of a lot more than society currently allows them to show. The COVID‐19 pandemic has changed the way society works. Our study has shown that for some adults with intellectual/developmental disabilities the digital world has opened possibilities that were not available to them before the pandemic. The pandemic has helped reinforce that thinking outside the box can allow adults with intellectual/developmental disabilities to demonstrate higher capabilities.
2. Being online has allowed the organisation to reach a wider group of people, including people who are more vulnerable. It can reduce the number of barriers to social inclusion that adults with intellectual/developmental disabilities commonly face when they are trying to participate in society.
3. Online events/activities take away some of the risks or unknown factors that come from being in the community. This allows people to put themselves out of their comfort zone while being in a safe place (i.e., their home). By doing this, adults with intellectual/developmental disabilities may be able to gain more confidence when out in community.
4. When you see someone that you relate to challenging themselves and taking on leadership roles it impacts what you think is possible for yourself.
5. Regular online connection with others, particularly those with whom you have shared experiences, are meaningful and important for mental wellbeing. Being part of a digital community helped adults with intellectual/developmental disabilities feel less socially isolated during a time of physical isolation.
6. The grassroots organisation was able to successfully provide leadership roles for adults with intellectual/developmental disabilities by providing support, deadlines, and training required for using online platforms and developing material for hosting events. Adults with disabilities are treated as equal partners of the organisation and their input and knowledge is valued, respected, and utilised by the organisation.

## Data Availability

The data that support the findings of this study are available on request from the corresponding author (Natasha A. Spassiani). The data are not publicly available due to (restrictions for example, their containing information that could compromise the privacy of research participants).
